# Systematic Review and Pragmatic Clinical Approach to Oral and Nasal Vitamin B12 (Cobalamin) Treatment in Patients with Vitamin B12 Deficiency Related to Gastrointestinal Disorders

**DOI:** 10.3390/jcm7100304

**Published:** 2018-09-26

**Authors:** Emmanuel Andrès, Abrar-Ahmad Zulfiqar, Khalid Serraj, Thomas Vogel, Georges Kaltenbach

**Affiliations:** 1 Department of Internal Medicine, Diabetes and Metabolic Diseases, Hôpitaux Universitaires de Strasbourg, 67091 Strasbourg, France; 2 Department of Geriatrics, CHRU de Rouen, 76000 Rouen, France; abzulfiqar@gmail.com; 3 Department of Internal Medicine, CHRU, d’Oujda 60049, Morocco; serrajkhalid@yahoo.fr; 4 Department of Geriatrics and Internal Medicine, Hôpitaux Universitaires de Strasbourg, 67091 Strasbourg, France; thomas.vogel@chru-strasbourg.fr (T.V.); georges.kaltenbach@chru-strasbourg.fr (G.K.)

**Keywords:** vitamin B12, Cobalamin, oral vitamin B12 treatment, nasal vitamin B12 treatment, Biermer’s disease, food-cobalamin malabsorption

## Abstract

The objective of this review is to provide an update on the effectiveness of oral and nasal vitamin B12 (cobalamin) treatment in gastrointestinal (GI) disorders. Relevant articles were identified by PubMed and Google Scholar systematic search, from January 2010 and June 2018, and through hand search of relevant reference articles. Additional studies were obtained from references of identified studies, the Cochrane Library and the ISI Web of Knowledge. Data gleaned from reference textbooks and international meetings were also used, as was information gleaned from commercial sites on the web and data from CARE B12 research group. For oral vitamin B12 treatment, 4 randomized controlled trials (vs. intramuscular), 4 narrative and 4 systematic reviews, and 13 prospective studies fulfilled our inclusion criteria. These studies concerned patients with vitamin B12 deficiency related to: food-cobalamin malabsorption (*n* = 6), Biermer’s disease (*n* = 3), veganism or vegetarianism (*n* = 1), total gastrectomy after Roux-en-Y gastric bypass (*n* = 2) and Crohn’s disease (*n* = 1). Four prospective studies include patients with vitamin B12 deficiency related to the aforementioned etiologies, except veganism or vegetarianism. The systematic present review documents that oral vitamin B12 replacement, at a daily dose of 1000 μg (1 mg), was adequate to normalize serum vitamin B12 levels and cure main clinical manifestations related to vitamin B12 deficiency, in GI disorders, and thus, with safety profile. For nasal vitamin B12 treatment, only one preliminary study was available. We conclude that oral vitamin B12 is an effective alternative to intramuscular vitamin B12 (except in patients presenting with severe neurological manifestations). Oral vitamin B12 treatment avoids the discomfort, contraindication (in patients with anticoagulation), and cost of monthly injections.

## 1. Introduction

Gastrointestinal (GI) disorders affect millions of people of all ages [1]. They are the most commonly presented GI illnesses seen by doctors in primary care, in internal medicine or in gastroenterology. The social and economic costs of GI disorders are enormous. The symptoms of these disorders can cause discomfort ranging from inconvenience to deep suffering or severe and even life-threatening manifestations [2]. Several of these GI disorders can lead to or be associated with a vitamin B12 (cobalamin) deficiency [3]. In this setting, the best-known GI disorders responsible for vitamin B12 (B12) deficiency are Biermer’s disease, also called pernicious anemia, and gastrectomy [4]. Therefore, the treatment of B12 deficiency is based on the parenteral administration of this vitamin [1,5].

Nevertheless in this setting, new routes of B12 replacement, particularly nasal and oral, have been developed [5]. In fact, between 1–5% of “free” or “crystalline” cobalamin is absorbed by passive diffusion along the entire GI tract, from the oral cavity or nasal mucosa to the colic mucosa [5]. For this, the treatment is based on B12 (cyano- and hydroxo-cobalamin) in the form of pills, tablets or oral solutions. Nevertheless to date, oral or nasal B12 replacement remains one of the “best secrets in medicine” [6].

This systematic review summarizes the current knowledge on the efficacy and safety of oral and nasal B12 (cobalamin) treatment in patients with cobalamin deficiency related to GI disorders.

## 2. Methodology of the Literature Search

A systematic literature search was performed on the PubMed database of the US National Library of Medicine and on Scholar Google. We searched for articles published between January 2010 and June 2018, using the following key words or associations: “gastrointestinal disorders”, “vitamin B12 deficiency”, “cobalamin deficiency”, “Biermer’s disease”, “pernicious anemia”, “gastrectomy”, “food-cobalamin malabsorption”, “*Helicobacter pylori*”, “atrophic gastritis”, “inflammatory bowel disease”, “exocrine pancreatic insufficiency”, “oral vitamin B12 therapy”, “oral cobalamin therapy”, “oral vitamin B12 treatment”, “oral cobalamin treatment”, “nasal vitamin B12 therapy”, and “nasal cobalamin therapy”; restrictions included: English- or French-language publications; published from 1 January 2010, to 1 July 2018; human subjects; adults and elderly subjects, clinical trials, review articles or guidelines.

Additional studies were obtained from references of identified studies, the Cochrane Library and the ISI Web of Knowledge. Data gleaned from international meetings were also used, as information gleaned from commercial sites on the web. American Society of Hematology educational books, textbooks of hematology and internal medicine were also used, as was information gleaned from international meetings and from commercial web sites.

All of the English and French abstracts were reviewed by at least two senior researchers from our research team (CAREnce en vitamine B12 [CARE B12], in the university hospital of Strasbourg, Strasbourg, France).

## 3. Results of the Literature Search

### 3.1. Synthetic Results of the Systematic Research

**Figure 1:**
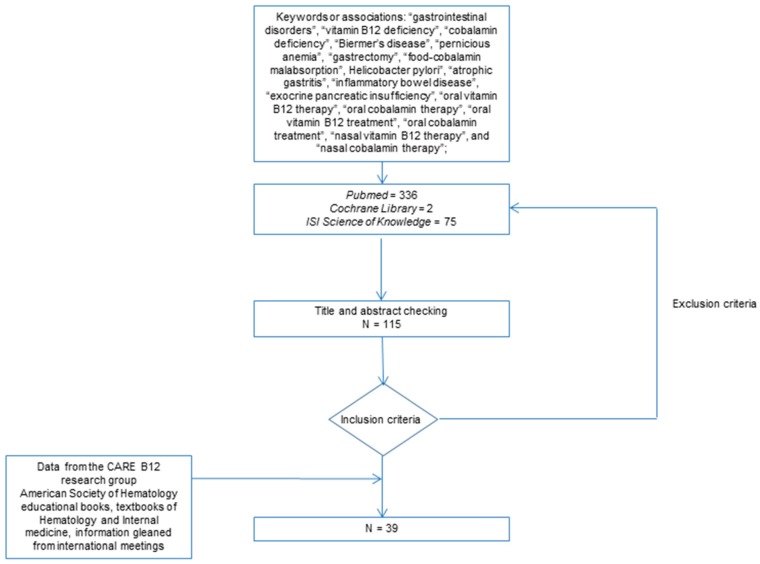
Flowchart of the reference research.

We reviewed 413 references, which yielded 115 potentially relevant papers (Figure 1). Forty-four papers met the original inclusion criteria. Of these, 39 contained sufficient published data to be included in this systematic review on oral and nasal vitamin B12 treatment in GI disorders [5,7,8,9,10,11,12,13,14,15,16,17,18,19,20,21,22,23,24,25,26,27,28,29]. The latter were used to write the present paper.

**-** For oral vitamin B12 treatment

Four prospective randomized controlled trials (vs. intramuscular vitamin B12 treatment) [7,8,9,10], four systematic reviews [11,12,13,14], six narrative reviews [5,15,16,17,18,19,20] and thirteen prospective studies fulfilled our inclusion criteria (Figure 1) [21,22,23,24,25,26,27,28,29,30,31,32,33,34]. These studies concerned patients with B12 deficiency related to a specific disorder or disease, e.g., food-cobalamin malabsorption (*n* = 6), Biermer’s disease (*n* = 3), veganism or vegetarianism (*n* = 1), total gastrectomy after Roux-en-Y gastric bypass (*n* = 2), and Crohn’s disease (*n* = 1). Four prospective studies include patients with B12 deficiency related to the aforementioned etiologies, except veganism or vegetarianism. All these studies included adults and elderly patients.

**-** For nasal vitamin B12 replacement

Only one preliminary well-documented study of nasal B12 treatment was available in elderly patients [35].

### 3.2. Randomized Controlled Studies of Oral Vitamin B12 Treatment in Vitamin B12 Deficiency

Four prospective randomized controlled studies comparing oral B12 vs. intramuscular (I.M.) B12 replacement have well-documented the efficacy and safety of oral B12 as a curative treatment (Table 1) [7,8,9,10]. In these studies, efficacy was defined as: the normalization and or a significant increase of the serum B12 level; improvements of hematological abnormalities and of neurological signs. These studies include patients with well-documented B12 deficiency related to GI disorders. It is worth noting that the treatment regime of B12 (frequency and daily dose) the oral and I.M. groups varied in these studies.

In a first study, Kuzminski et al., in a prospective randomized trial including 38 patients, reported improvement of hematological parameters and B12 levels (mean value: 907 pg/mL), after four months of oral cyanocobalamin replacement using a much higher dose (i.e., 2000 µg per day (2 mg)) (Table 1) [7]. In this study, serum B12 levels were significantly higher in the oral (B12 at a daily dose of 2000 μg (2 mg) compared with I.M. (B12 at a daily dose of 1000 μg (1 mg) group at two months: 643 ± 328 vs. 306 ± 118 pg/mL; *p* < 0.001. The difference was even greater at four months: 1005 ± 595 vs. 325 ± 165 pg/mL. Four of the 18 patients in the oral group and 4 of the 15 in the I.M. group had a neurological response with a marked improvement or clearing of paresthesia, ataxia, or memory loss.

Bolaman et al., in a prospective randomized trial of 60 patients, also reported significant improvement of hematological parameters and B12 levels (mean improvement: +140.9 pg/mL), after three months of daily 1000 µg of oral cyanocobalamin treatment (Table 1) [8]. In this study, there was an increase in serum B12 levels in both groups (oral B12 at 1000 μg (1 mg) vs. I.M. vitamin B12 at 1000 μg) at 90 days: 213.8 pg/mL in the oral and 225.5 pg/mL in the I.M. group. There was a statistically significant difference between days 0 and 90 in both groups (*p* < 0.0001), but authors did not analyze difference between both groups. Both groups reported improvements of cognitive functions, sensory neuropathy, and vibration sense, but there was no statistical significant difference between both groups.

In a controlled, randomized, multicenter, parallel, non-inferiority clinical trial (OB12 study) lasting one year, a preliminary analysis gives a glimpse that the oral route for B12 replacement may be as effective and safe and administered at a lower cost than the I.M. route (ongoing study) (Table 1) [9]. This study involves 23 primary healthcare centers in Spain and must include 320 patients ≥ 65 years of age. In this study, I.M. B12 will be administered as follows: 1000 µg on alternate days in weeks 1 and 2, 1000 µg per week in weeks 3–8, and 1000 µg per month in weeks 9–52. In the oral arm, the B12 will be administered as: 1000 µg per day in weeks 1–8 and 1000 µg per week in weeks 9–52. At the time of writing, the study was ongoing.

In another randomized, parallel-group, double-blind, dose-finding trial, Eussen et al. showed that the lowest dose of oral cyanocobalamin required to normalize mild B12 deficiency is more than 200 times the recommended dietary allowance of approximately 3 µg daily (i.e., >500 µg (0.5 mg) per day) (Table 1) [10]. Apart from helping to determine the dose, this study gives no indication for the practitioners of the clinical effectiveness of oral B12 treatment.

It is to note that the effect of oral B12 treatment in patients presenting with ‘severe’ neurological manifestations (e.g., combined sclerosis) has not yet been adequately documented in these studies [15]. Thus until this has been studied, parenteral B12 replacement (mainly I.M.) is still to be recommended for such patients.

To our knowledge, only two of the aforementioned trials studied oral B12 replacement reported data on adverse events, with two of such adverse events (allergic cutaneous reaction) in one study in the group of oral vitamin B12 [7,8].

### 3.3. Systematic Reviews of Oral Vitamin B12 Treatment in Vitamin B12 Deficiency

The first evidence-based analysis by the Vitamin B12 Cochrane Group in 2005 [11], as well two other analyses [12,14], support the efficacy of oral B12 as a curative treatment, with a dose between 1000 and 2000 µg initially prescribed daily and then weekly. These systematic reviews include patients with well-documented B12 deficiency related to GI disorders (e.g., Biermer’s disease, food-cobalamin malabsorption) [11,12,14]. In these analyses, serum B12 levels increased significantly in patients receiving oral B12 and both groups of patients (receiving oral and I.M. B12 treatment) showed an improvement in neurological symptoms (not well-defined).

In this setting, the Cochrane Group concludes that: “daily oral replacement may be as effective as I.M. administration in obtaining short term hematological and neurological responses in vitamin B12 deficient patients” [11]. The review from the CARE B12 team leads to the same conclusion but excludes severe psycho-neurological manifestations (e.g., combined sclerosis) of the indication of oral B12 replacement [14].

The second evidence based-analysis from the same aforementioned group in 2018 confirms their previous analysis [13]. In fact, the Cochrane Group concludes that: “there is evidence of low quality that oral vitamin B12 or vitamin B12 administered intramuscularly have similar effects in normalizing vitamin B12 serum levels, but oral treatment costs less. Further trials should conduct better randomization and blinding procedures, recruit more participants, and provide adequate reporting. Future trials should also measure important outcomes such as the clinical signs and symptoms of vitamin B12 deficiency, health related-quality of life, socioeconomic effects, and report adverse events adequately.”

### 3.4. Prospective Studies of Oral Vitamin B12 Treatment in Patients with Food-Cobalamin Malabsorption and Biermer’s Disease

**Figure 2:**
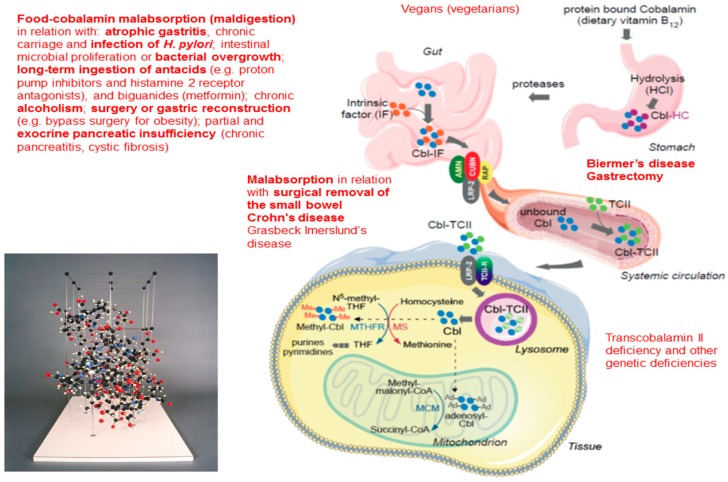
Structure and metabolism of the vitamin B12, with a focus on gastrointestinal disorders responsible for vitamin B12 deficiency. The digestive step of the metabolic of cobalamin (cbl) begins with nutrient intake to its intestinal absorption. Endocytic receptors and proteins responsible for vitamin B12 intestinal absorption include cubilin (CUBN), amnionless (AMN), receptor-associated protein and megalin (MGA1). The membrane megalin/transcobalamin II (TCII)-receptor complex allows the cellular uptake of cbl. Lysosomal-mediated degradation of TCII and subsequent release of free-cbl is essential for vitamin B12 metabolic functions. MS, methonine synthase; THF, tetrahydrofolate; MTHFR, methylene tetrahydrofolate reductase; MCM, methylmalonyl CoA mutase.

Our working CARE B12 group has developed an effective oral curative treatment for patients presenting with GI disorders: food-cobalamin malabsorption (maldigestion, FCM) and Biermer’s disease, using crystalline cyanocobalamin (Figure 2) [21,22,23,24,25]. This treatment has been validated through several studies on small numbers of patients with a well-documented B12 deficiency, this latter in relation with well-documented disorders (Table 2 and Table 3). In these studies, the FCM is related to several GI disorders, e.g., mainly atrophic gastritis, chronic carriage and infection of *H. pylori*, long-term ingestion of proton pump inhibitors, chronic alcoholism, and partial and exocrine pancreatic insufficiency [21,22,23,24,25]. In the majority of studies, the effectiveness of treatment has been validated on the correction of serum B12 levels and that of hematological abnormalities (primary criteria), in general at three months (short term efficacy) [21,22,23,24,25]. In some studies, correction of neurological signs or clinical manifestations (e.g., dysesthesia, asthenia) has also been sought (secondary criteria). In all the studies, adverse events were studied.

In a first study, the CARE B12 group prospectively studied 10 patients with well-established B12 deficiency and FCM who received 3000 or 5000 µg of oral crystalline cyanocobalamin once a week, for at least three months (Table 2) [21]. After three months of oral B12 replacement, all patients had increased hemoglobin levels (mean increase of 1.9 g/dL; 95% confidence interval: 0.9 to 3.9 g/dL; *p* < 0.01 compared with baseline), and decreased mean erythrocyte cell volume (mean decrease of 7.8 fL; 95% confidence interval: 0.9 to 16.5 fL; *p* < 0.001). Two patients had only minor, if any, responses. Serum B12 levels (primary criteria) were increased in all eight patients in whom it was measured.

Table 2 describes the other studies conducted on oral vitamin B12 treatment (open, not randomized studies) by the CARE B12 group [22,23,24]. Analysis of these data confirms the previously reported efficacy of oral crystalline cyanocobalamin; almost exclusively in elderly patients with FCM (see the causes of the FCM higher in the text). All of the patients who were treated orally corrected their B12 levels (primary criteria) and at least two-thirds corrected their hematological abnormalities. Moreover, one-third of patients experienced a clinical improvement on oral B12 replacement (secondary criteria). In most cases of FCM a low B12 dose (i.e., 125–1000 µg (0.125–1 mg) of oral crystalline cyanocobalamin per day) was used.

The aforementioned results were also observed in a population of patients presenting with Biermer’s disease (Table 3). In this setting, the CARE B12 group studied in an open study 10 patients with well-documented B12 deficiency related to Biermer’s disease who daily received 1000 µg of oral crystalline cyanocobalamin for at least three months [25]. After three months of treatment, serum B12 levels (primary criteria) were increased in all nine patients in whom it was measured (mean increase of 117.4 pg/mL; *p* < 0.0000003 compared with baseline). Eight patients had increased hemoglobin levels, with a mean increase of 2.45 g/dL; *p* < 0.01. All 10 patients had decreased mean erythrocyte cell volume (mean decrease of 10.4 fL; *p* < 0.003). Three patients (one-third) experienced clinical improvements (secondary criteria).

Two other studies have documented the efficacy of oral B12 treatment in patients with Biermer’s anemia (Table 3) [26,27]. These studies also had small sample size but had longer follow-up period (up to 18 months). Oral B12 was effective in all the patients (in which 10 patients had Biermer’s disease) in Nyholm’s study (*n* = 40), with the median serum B12 level of 1193 pg/mL after three months of oral B12 replacement [26]. It was also reported that using oral B12 treatment did not result in any new neurological complications. Normalization of serum B12 levels was seen in all patients (inclusive of patients with pernicious anemia) in Delpre’s study with oral (sublingual) B12 replacement (*n* = 18) [27]. An increase in B12 level was as much as fourfold compared with pre-treatment in most patients. The mean change of 387.7 pg/mL was significant (*p* = 0.0001).

The CARE B12 group had also documented the long-term efficacy of oral B12 treatment (with a mean daily dose of cyanocobalamin of 500 µg (0.5 mg)), with a median follow up of 2.5 years, in a population of 22 patients with well-established cobalamin deficiency (Table 2) [28]. These preliminary findings are in accordance with the results of Roth’s study, with a median follow up of more than four years on oral B12 replacement [29]. The CARE B12 group also documented in a small study (10 patients with FCM and Biermer’s disease) the relative efficacy of oral cyanocobalamin treatment on cognitive functions (mini mental state examination score) (Table 2) [30]. In the CARE B12 studies including 132 patients [21,22,23,24,25,30], only 2 of these (1.5%) reported treatment-related adverse events of the oral replacement, such as skin allergy.

### 3.5. Oral Vitamin B12 Treatment in Vegans or Vegetarians

In vegans and vegetarians with a marginal deficiency (*n* = 40), Del Bo’ C et al. studied in a randomized controlled trial the effect of two different sublingual dosages of B12 on cobalamin nutritional status [31]. Their results support the use of a sublingual dosage of 50 μg per day (350 μg (0.35 mg) per week) of vitamin B12, instead of 2000 μg (2 mg) per week (provided as a single dose).

### 3.6. Oral Vitamin B12 Treatment in Patients with Total Gastrectomy after Roux-en-Y Gastric Bypass

In patients submitted to total gastrectomy (*n* = 26), Moleiro et al. performed a prospective uncontrolled study in order to evaluate the clinical and laboratory efficacy of long-term oral B12 replacement (1000 µg (1 mg) per day) [32]. Patients were included with a mean period of 65 months (3–309) after total gastrectomy. During follow-up (mean: 20 months (8.5–28)), all patients had normal B12 levels. The patient with low B12 levels at inclusion had an increase to adequate levels, which remained stable. There were no differences with statistical significance among B12 levels at 6 (867 pg/mL), 12 (1008 pg/mL), 18 (1018 pg/mL), and 24 (1061 pg/mL) months.

In patients submitted after Roux-en-Y gastric bypass (RYGB) (*n* = 50), Schijns et al. investigate whether oral methylcobalamin (1000 µg (1 mg) per day) supplementation increases and normalizes low B12 concentrations in RYGB patients as compared to I.M. hydroxocobalamin injections [33]. At six months, B12 levels normalized in all individuals, and there was no significant difference in B12 between the two groups. Methyl malonic acid (MMA) and homocysteine concentrations decreased significantly after six months within each group (*p* < 0.001 and *p* < 0.001 for MMA and *p* = 0.03 and *p* = 0.045 for homocysteine, respectively). There was no significant difference between the groups at six months for both MMA and homocysteine (*p* = 0.53 and *p* = 0.79).

### 3.7. Oral Vitamin B12 Treatment in Patients with Crohn’s Disease

In a multicenter retrospective cohort study that included 94 patients with Crohn’s disease (including 24 patients with ileal resection) and B12 deficiency, Gomollón et al. studied the efficacy of oral B12 treatment [34]. In this setting they reported that 94.7% of the patients normalized their B12 levels with oral B12 replacement (most used dose of 1000 µg (1 mg) per day). After a mean follow-up of three years, the oral route was effective as maintenance treatment in 81.7% of patients. A lack of treatment adherence was admitted by 46.6% of patients in who the oral route failed.

### 3.8. Oral Vitamin B12 Treatment in a Reference Academic Center

Since the 1990s, at least half of the patients followed in the Hôpitaux Universitaires de Strasbourg (University Hospital of Strasbourg, Strasbourg, France) with well-documented B12 deficiency were treated with oral cyanocobalamin, with a dose between 125 and 2000 µg (0.125 and 2 mg) per day [14]. In the Department of Internal Medicine in the aforementioned Institution (>800 followed patients with a documented cobalamin deficiency, median age 71 years), FCM accounts for about 60–70% of the cases of B12 deficiency in elderly patients, whereas Biermer’s disease accounted for only 15–25%. All of these patients who were treated orally corrected their B12 levels and at least 80% corrected their hematological abnormalities. Moreover, half of the patients experienced a clinical improvement on oral B12 treatment. It is to note that the patients presenting with severe neurological manifestations were usually excluded by our team for the oral B12 replacement. In the experience of the CARE B12 group, oral B12 replacement avoids the discomfort, inconvenience (contraindication in patients under long-term anticoagulant and/or platelet anti-aggregate agents), and perhaps cost of monthly injections (I.M. B12 replacement required a nurse).

In clinical practice, one of the predominant elements for the choice of treatment options is the patient preference [19,20]. This latter should absolutely be taken into consideration. In our experience, other factors of this choice (I.M. B12 vs. oral B12 replacement) include patient compliance and patient comorbidities. Thus in patients with non-compliance to oral medication (E.G. cognitive impairment), I.M. route may be a better option to ensure timely administration. On the other hand, oral replacement may improve adherence for patients who prefer oral medication to injections or present contraindication (e.g., coagulopathy). In this setting, surveys on patients’ preferences for oral or I.M. B12 replacement may be informative to guide clinical decision-making.

### 3.9. Nasal Vitamin B12 Treatment

Historically, other pathways than the oral and the I.M. route (more rarely intravenous) have also been developed in the setting of B12 deficiency, as the nasal route [35]. This route of administration of B12 has seen a revival of interest in recent years [36,37,38,39]. This renewed interest is mainly due to the development of several commercial forms of intra-nasal spray-dried powders or nasal gel of cyanocobalamin. These latter (e.g., 500 μg/0.1 mL administered intra-nasally once weekly for Nascobal^®^) have been approved as treatment for B12 deficiency, including pernicious anemia [38].

To our knowledge, only one recent study (quiet of poor methodological quality) has documented the clinical utility of the nasal vitamin B12 replacement, with regards to pharmacokinetics, efficacy, and safety [39]. In this study, the estimated cobalamin bioavailability after intranasal administration was 2%.

## 4. Conclusions and Recommendations

The present analysis support the use of oral B12 replacement in patients with B12 deficiency related to GI disorders, especially FCM (e.g., related to atrophic gastritis, *H. pylori* infection, gastric bypass), malabsorption (e.g., small intestinal resection), and Biermer’s disease (pernicious anemia). Nevertheless, oral B12 replacement remains to our experience uncommon in in clinical practice of everyday life. Thus to our opinion, it may be time to communicate on this topic and time to propose international recommendations to better convince clinicians on the feasibility and interest of oral B12 replacement [14].

**Figure 3:**
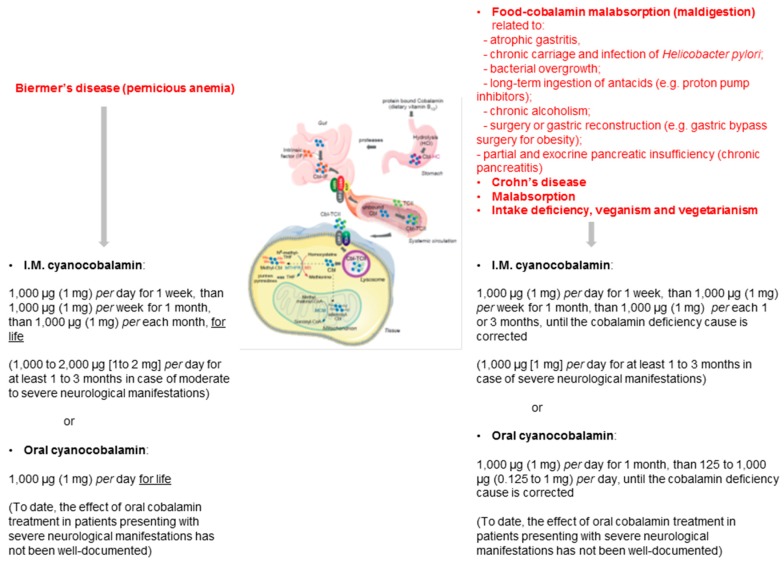
Pragmatic clinical approach to oral vitamin B12 treatment in patients with vitamin B12 deficiency related to gastrointestinal disorders [14,40].

In light of the present systematic review and personal experience, a pragmatic clinical approach may be proposed as: A dose of 1000 µg per day (1 mg per day) of oral cyanocobalamin for life in case of Biermer’s disease; A dose of 1000 µg per day of oral cyanocobalamin for 1 month and then a dose of 125 to 1000 µg (0.25 to 1 mg per day) per day in case of intake deficiency or food-cobalamin malabsorption, until the cobalamin deficiency cause is corrected (Figure 3) [14,40].

In this context, ongoing oral B12 replacement may be necessary until any associated GI disorders are corrected, if possible: e.g., by halting the ingestion of the offending medication or alcoholism; by treating *H. pylori* infection until the germ eradication is documented; or by supplemented pancreatic exocrine failure [14]. This may result in lifelong replacement or, when applicable, sequential administration. For all doctors, it is important to keep in mind that Biermer’s disease is a predisposing condition for all types of cancers of the stomach, which may require endoscopic surveillance [4].

In our opinion, patients with B12 deficiency who are symptomatic have severe neurological deficits or have critically low blood levels of B12 should be treated with I.M. B12 replacement. This is to ensure rapid replenishment of body stores to prevent irreversible consequences of the cobalamin deficiency. Subsequently, patients may be able to convert to oral replacement with close monitoring [14].

This alternative route of replacement had been developed and proposed as a way of avoiding the discomfort, inconvenience, and cost of monthly injections (with the need of a nurse) [29,30]. In practice, they require a strict treatment observance of the patient [31]. For nasal B12 treatment, further studies seem necessary to use this route in clinical practice, especially in patients with documented B12 deficiency.

### Perspectives and Direction of Future Research

To date, the oral B12 treatment regimen is not yet formally approved. Further studies should include testing the efficacy of different molecules (cyano-, hydroxo-, methyl-cobalamin) and dosages. In this setting, it may be important to study the knowledge and practices of doctors/health-care workers with regard to oral B12 replacement for patients with documented cobalamin deficiency.

To date, recent developments in conjunction with nanomedicine for the co-administration of drugs with lipid compounds have been reported to enhance lymphatic transport [41]. These technologies have been recently used to administrate vitamin B12. Future developments are expected in this field

Sublingual administration of vitamin B12 (placing the vitamin beneath the tongue for one to two minutes) is another promising way of administration which has been studied historically [27] and which is currently experiencing renewed interest.

## Figures and Tables

**Table 1:** Prospective randomized controlled studies of oral vitamin B12 treatment (number of studies = 4).

Study Characteristics (Number of Patients)	Therapeutic Modalities	Results
Prospective randomized controlled study including patients with vitamin B12 deficiency related to Biermer’s disease, malabsorption and maldigestion (number of patients (*n*) = 38) [7].	Oral crystalline cyanocobalamin: 2000 µg per day, for at least four months (“oral group”) vs. standard treatment with I.M. cyanocobalamin.	- The mean pre-treatment values for serum vitamin B12, methylmalonic acid (MMA), and homocysteine were, respectively, 93 pg/mL, 3850 nmol/L, and 37. 2 µmol/L in the “oral group” and 95 pg/mL, 3630 nmol/L, and 40.0 µmol/L in the “I.M. group”. After four months of therapy, the respective mean values were 1005 pg/mL, 169 nmol/L, and 10.6 µmol/L in the “oral group” and 325 pg/mL, 265 nmol/L, and 12.2 µmol/L in the “I.M. group”. The higher serum vitamin B12 and lower serum MMA levels at four months post-treatment in the “oral group” vs. the “I.M. group” were significant, with *p* < 0.0005 and *p* < 0.05, respectively. - Correction of hematological and neurological abnormalities was prompt and indistinguishable between the two groups.
Prospective randomized open-label study including patients with vitamin B12 deficiency related to Biermer’s disease, and food-cobalamin malabsorption (*n* = 60) [8].	Oral crystalline cyanocobalamin: 1000 µg, once daily for 10 days (“oral group”) or cobalamin I.M.: 1000 µg once daily for 10 days (“I.M. group”). After 10 days, both treatments were administered once a week for four weeks, and after that, once a month for life.	- The mean serum vitamin B12 concentration increased significantly from day 0 to 90 (*p* < 0.001). - In the “oral group”, at days 30 and 90, all hematological parameters changed significantly vs. day 0 (mean hemoglobin levels increased (both *p* < 0.001); mean corpuscular volume decreased (both *p* < 0.001); mean white blood cell count increased (day 30, *p* < 0.01; day 90, *p* < 0.001); and mean platelet count increased (both *p* < 0.001)). Reticulocytosis was observed in all patients. These hematological parameters and the recovery patterns were similar between the two groups. - Neurological improvement was detected in 78% in the “oral group” and 75% in the “I.M. group” at day 30.
Randomized, parallel-group, double-blind, dose-finding trial including patients with vitamin B12 deficiency from not determined cause (*n* = 120) [10].	Daily oral doses of 2.5, 100, 250, 500, and 1000 µg of cyanocobalamin administered for 16 weeks.	- The lowest dose of oral cyanocobalamin required to normalize mild vitamin B12 deficiency is more than 200 times the recommended dietary allowance of approximately 3 µg daily (i.e., >500 µg per day).
Controlled, randomized, multicenter, parallel, non-inferiority clinical trial (*OB12* study) 23 primary healthcare centers in Spain (*n* = 350) [9].	‘I.M. vitamin B12 group’: 1000 µg on alternate days in weeks 1 and 2, 1000 µg per week in weeks 3–8, and 1000 µg per month in weeks 9–52 vs. “oral group”: 1000 µg per day in weeks 1–8 and 1000 µg per week in weeks 9–52.	- Ongoing study. - Preliminary results seem to show the same clinical benefit in the two groups.

**Table 2:** Studies of oral vitamin B12 treatment in patients with vitamin B12 deficiency related to food-cobalamin malabsorption (maldigestion) in relation mainly with atrophic gastritis, chronic carriage and infection of *Helicobacter pylori*, bacterial overgrowth, long-term ingestion of antacids (e.g., proton pump inhibitors), chronic alcoholism, surgery or gastric reconstruction (e.g., bypass surgery for obesity), and partial and exocrine pancreatic insufficiency (chronic pancreatitis) (number of studies = 6).

Study Characteristics (Number of Patients)	Therapeutic Modalities	Results
Open prospective study of vitamin B12 deficiency related to food-cobalamin malabsorption (*n* = 10) [21].	Oral crystalline cyanocobalamin: 650 µg per day, for at least three months.	- Normalization of serum vitamin B12 levels in 80% of the patients. - Significant increase in Hb levels (mean of 1.9 g/dL) and decrease of mean ECV (mean of 7.8 fL). - Improvement of clinical abnormalities in 20% of the patients. - No adverse effect.
Open prospective study of vitamin B12 deficiency related to food-cobalamin malabsorption (*n* = 30) [22].	Oral crystalline cyanocobalamin: between 1000 µg and 250 µg per day, for one month.	- Normalization of serum vitamin B12 levels in 87% of the patients. - Significant increase of Hb levels (mean of 0.6 g/dL) and decrease of ECV (mean of 3 fL); normalization of Hb levels and ECV in 54% and 100% of the patients, respectively. - Dose effect—effectiveness dose of vitamin B12 at a dose of 500 µg per day. - No adverse-effect.
Open prospective study of low vitamin B12 levels not related to pernicious anemia (*n* = 20) [23].	Oral crystalline cyanocobalamin: between 1000 µg per day for at least one week.	- Normalization of serum vitamin B12 levels in 85% of the patients. - No adverse-effect.
Open prospective study of low vitamin B12 levels not related to pernicious anemia (*n* = 30) [24].	Oral crystalline cyanocobalamin: between 1000 µg and 125 µg per day for at least one week.	- Normalization of serum vitamin B12 levels in all patients with at least a dose of vitamin B12 at a dose of 250 µg per day. - Dose effect—effectiveness dose of vitamin B12 at a dose of 500 µg per day. - No adverse-effect.
Cohort study of low vitamin B12 levels mainly related to food-cobalamin malabsorption (*n* = 22) [28].	Oral crystalline cyanocobalamin: 650 µg per day, for a median of 2.5 years.	- Normalization of serum vitamin B12 levels in 95% of the patients. - Significant increase of Hb levels (mean of 1.1 g/dL). - Improvement of clinical abnormalities in 20% of the patients.
Cohort study of patients with cognitive alteration related to low vitamin B12 levels mainly related to food-cobalamin malabsorption (*n* = 10) [30].	Oral crystalline cyanocobalamin: 1000 µg per day, for a week, then 1000 µg per week, for a month, and 1000 µg per month, for at least three months.	- Increase of MMSE score during the treatment (*p* < 0.06).

Hb = hemoglobin. ECV = erythrocyte cell volume. MMSE = mini mental state examination.

**Table 3:** Studies of oral vitamin B12 treatment in patients with vitamin B12 deficiency related to Biermer’s disease (pernicious anemia) (number of studies = 3).

Study Characteristics (Number of Patients)	Therapeutic Modalities	Results
Open prospective study of low vitamin B12 levels related to pernicious anemia (*n* = 10) [25].	Oral crystalline cyanocobalamin: 1000 µg per day, for at least 3 months.	- Significant increase of serum vitamin B12 levels in 90% of the patients (mean of 117.4 pg/mL). - Significant increase of Hb levels (mean of 2.45 g/dL) and decrease of ECV (mean of 10.4 fL). - Improvement of clinical abnormalities in 30% of the patients.
Prospective, case series of low vitamin B12 levels (*n* = 40), including 10 patients with Biermer’s disease [26].	Loading dose of IM vitamin B12 till vitamin B12 level reached lower 25th centile (418 pg/mL) and then converted to oral vitamin B12 1000 μg per day, for 3–18 months.	- Normalization of the serum vitamin B12 levels in all patients. At three months, median serum vitamin B12 level of 1193 pg/mL. - No adverse effect.
Open prospective study of low vitamin B12 levels related to Biermer’s disease (*n* = 18) [27].	Sublingual cobalamin for 7–12 days.	- Normalization of serum vitamin B12 levels. Significant mean change of 387.7 pg/mL (*p* = 0.0001). - Increase in vitamin B12 level as much as fourfold compared with pre-treatment in most patients.

Hb = hemoglobin. ECV = erythrocyte cell volume.
